# Infiltrating mast cells enhance benign prostatic hyperplasia through IL-6/STAT3/Cyclin D1 signals

**DOI:** 10.18632/oncotarget.19465

**Published:** 2017-07-22

**Authors:** Zhenyu Ou, Yao He, Lin Qi, Xiongbin Zu, Longxiang Wu, Zhenzhen Cao, Yuan Li, Longfei Liu, Daud Athanasius Dube, Zhi Wang, Long Wang

**Affiliations:** ^1^ Department of Urology, Xiangya Hospital, Central South University, Changsha, China; ^2^ Department of Gynecologic Oncology, The Affiliated Tumor Hospital of Xiangya Medical School, Central South University, Changsha, China; ^3^ Department of Urology, College of Health Sciences, University of Zimbabwe, Harare, Zimbabwe

**Keywords:** benign prostatic hyperplasia, mast cell, proliferation, chemokine

## Abstract

Early evidences have showed that mast cells could infiltrate into benign prostatic hyperplasia (BPH) tissues, but the exact role of mast cells in BPH development remains unclear. In this study, we identified more mast cells existing in human BPH tissues compared with that in the normal prostate. In the *in vitro* co-culture system, BPH-1 prostate cells promoted activation and migration of mast cells, and mast cells conversely stimulated BPH-1 cells proliferation significantly. Molecular analysis demonstrated that mast cell-derived interleukin 6 (IL-6) could activate STAT3/Cyclin D1 signals in BPH-1 cells. Blocking IL-6 or STAT3 partially reverse the capacity of mast cells to enhance BPH-1 cell proliferation. Our findings suggest that infiltrating mast cells in BPH tissues could promote BPH development via IL-6/STAT3/Cyclin D1 signals. Therefore, targeting infiltrating mast cells may improve the therapeutic effect of BPH.

## INTRODUCTION

Benign prostatic hyperplasia (BPH) is the most common proliferative disease among elderly males [1]. There are several theories such as increased oxidative stress [1], imbalance of androgen/estrogen [2, 3], chronic inflammation [4] and metabolic syndromes [5] may explain the pathogenesis of BPH. However, the exact etiopathogenesis of this disorder is still unclarified.

Accumulating evidences have indicated that infiltrating inflammatory cells play an important role in BPH development. Histological analysis of surgically removed BPH specimens revealed that the components of the infiltrating cells included T lymphocytes, B lymphocytes, macrophages, as well as mast cells [6, 7]. Consistently, histological analysis of an animal model demonstrated that there were significantly increased mast cells infiltrated into benign prostatic hyperplasia tissues [8], implicating a potential role of mast cells in enhancing BPH development and progression. Previous studies have revealed that mast cells were involved in many pathophysiological processes, including fibrosis, tissue remodeling, chronic inflammation, and angiogenesis [9–12]. However, the dynamic cellular interaction between mast cells and benign prostatic hyperplasia components has not been well studied. The exact function of mast cells in this disorder remains unclear.

Interleukin 6 (IL-6) is a pleiotropic cytokine implicated in inflammation, infection responses, hematopoiesis, and malignant diseases. Molecular analysis of prostate tissue specimens revealed that IL-6 could activate important signaling pathways, such as Janus family tyrosine kinase (JAK)-signal transducer and activator of transcription (STAT) pathway, as well as the extracellular signal-regulated kinase 1 and 2 (ERK1/2)-mitogen-activated protein kinase (MAPK) pathway [13]. STAT-3 is the most commonly involved STAT family member in IL-6 signaling. IL-6 has been found, in an autocrine/paracrine manner, to exert its effects on cell growth, differentiation, and survival signals by activating STAT3 [14–16]. CyclinD1, a major component of cyclins, plays an important role in cell cycle progression [17]. As a transcription factor, STAT3 could bind to the cyclin D1 promoter and regulate its expression to regulate cell growth [18].

In the present study, we investigated the cross-talk between mast cells and benign prostatic hyperplasia epithelial cells. Our results indicated that benign prostatic hyperplasia epithelia cells could enhance mast cell migration and induce its activation. Conversely, mast cell-derived cytokine IL-6 promotes BPH-1 proliferation through STAT3/Cyclin D1 pathway. These findings demonstrated that the interaction between mast cells and benign prostatic hyperplasia epithelia cells contributes to the development of BPH and may help us develop better potential therapies for BPH.

## RESULTS

### Increased infiltrated mast cells in human BPH prostate tissues

**Figure 1:**
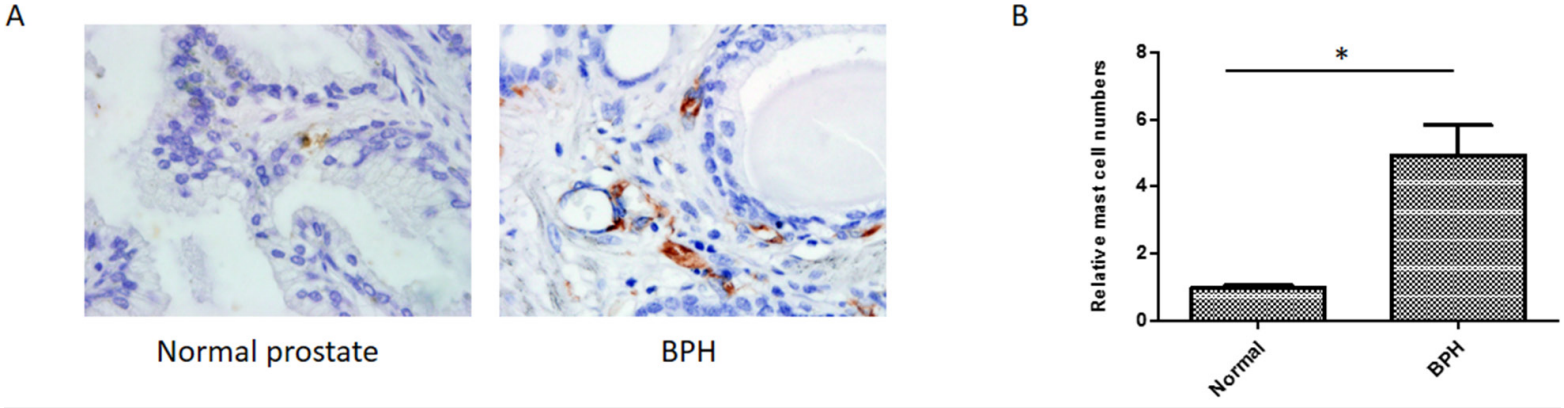
Immunohistochemical staining of human normal prostates and BPH tissues using anti-tryptase antibody **(A)** Paraffin-embedded tissue sections were used and representation specimens are shown (X400). **(B)** Quantitation of the IHC staining. *P < 0.05 by Student's t-test.

Previous studies indicated that infiltrated mast cells may contribute to BPH development and progression [8, 19]. To investigate whether the number of infiltrated mast cells is increased in human BPH specimens compared to that in normal human prostate tissues, we performed IHC on human BPH and normal prostate tissues with anti-tryptase antibody. Our results showed that more mast cells were identified in BPH tissues compared to the normal controls (Figure 1). This finding implies that infiltrated mast cells may affect the BPH development and progression.

### Increased mast cell migration and activation in a BPH-1 cells/mast cells co-culture system

**Figure 2:**
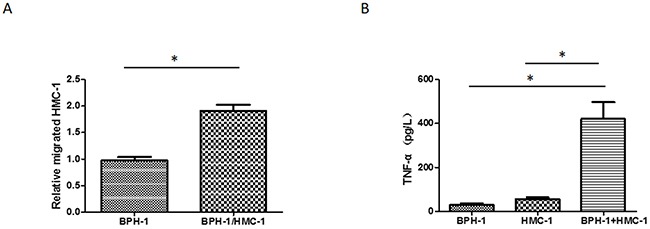
Mast cell migration and activation induced by conditioned medium from co-culuted BPH-1 cells/mast cells **(A)** HMC-1 cells were seeded into upper chambers of a transwell, and conditioned medium from BPH-1/HMC-1 or BPH-1 cells was added in to the lower chambers. Results are the quantitation of the relative migrated cells number of three independent experiments. **(B)** HMC-1 cells were cultured alone or with BPH-1 cells for 24 hours; supernatants were collected, and TNF-α release was determined by ELISA. Graphs shown are mean of three experiments.*P < 0.05 by Student's t-test.

We established a co-culture model to investigate the impact of BPH-1 cells on mast cells. The human mast cell line-1 (HMC-1) cells, derived from a patient with mast cell leukaemia, is an established cell line exhibiting a phenotype similar to that of human mast cells [20]. We seeded the HMC-1 cells in the upper chamber of the transwell system, added conditioned medium into the lower chamber and quantified migrated HMC-1 cells 6 hours later. We found that the co-cultured medium from BPH-1/HMC-1 cells could enhance the migration ability of HMC-1 cells comparing to the control medium from BPH-1 cells alone (Figure 2A).

To determine whether the cross-talk between benign prostatic hyperplasia prostate epithelium cells and infiltrating mast cells plays any role in activating the mast cells, we detected the TNF-α concentration in the conditioned medium. As reported previously, TNF-α release is a well-accepted method to measure HMC-1 activation [21]. The results showed that co-culturing the BPH-1 cells with HMC-1 cells led to a significantly increased TNF-α releasing (Figure 2B).

These data suggested that some factors which could promote mast cells activation and migration might be released into the conditioned medium during the cross-talk between BPH-1 and HMC-1 cells.

### BPH-1 cells could recruit more mast cells via CXCL12 expression

**Figure 3:**
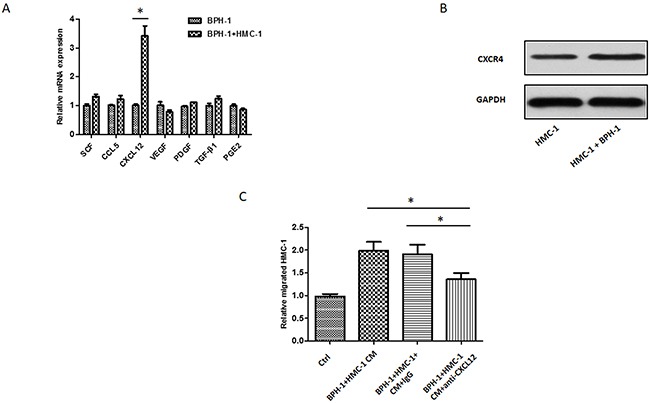
CXCL12/CXCR4 axis is responsible for BPH-1 induced mast cells migration **(A)** qRT-PCR screening of a panel of cytokine/chemokine factors that could be responsible for BPH-1 cell promoted mast cell migration. **(B)** Western blot shows the expression of CXCL12 receptor CXCR4 in mast cells. **(C)** The interruption assay by adding neutralizing antibody to CXCL12 in the migration system. The results show the relative migrated HMC-1 cells in different groups.*P < 0.05 by Student's t-test.

To explore why the interaction between BPH-1 cells and mast cells could promote mast cells migration, we conducted qRT-PCR assays to examine the potential cytokines/chemokines that might be related to the mast cell recruitment. As shown in Figure 3A, we identified that CXCL12 expression was significantly elevated in BPH-1 cells after co-culturing with the mast cells. We speculated that BPH-1 cells secreted CXCL12 to enhance mast cells migration. We further detected that the CXCL12 receptor CXCR4 expression in the mast cells. These results were in line with previous study [22], and the mast cells expressed CXCR4 and the co-culturing with BPH-1 cells could further increase its expression (Figure 3B). Furthermore, we observed that blocking CXCL12 function with neutralizing anti-CXCL12 antibodies resulted in partial inhibition of mast cells migration (Figure 3C). These results demonstrated that BPH-1 cells might recruit more mast cells to hyperplasia area via increasing the expression of CXCL12.

### Mast cells promoted BPH-1 cell proliferation

**Figure 4:**
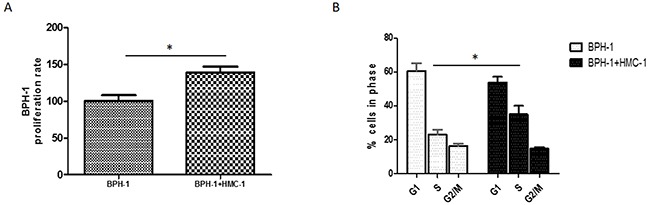
Proliferative effect of mast cells on BPH-1 cells **(A)** BPH-1 cells were cultured alone or co-cultured with mast cells and assayed with MTT at 72 hours. **(B)** Cell-cycle stage were assessed using PI staining and flow cytometry in BPH-1 cells with or without co-culturing with mast cells. Graphs shown are mean of three experiments. *P < 0.05 by Student's t-test.

To further investigate the role of mast cells in the pathogenesis of BPH, we examined whether the mast cells could affect BPH-1 cells proliferation. We applied MTT assay to measure the BPH-1 cells growth after co-culture with mast cells. The MTT results indicated that mast cells could increase BPH-1 cells proliferation significantly (Figure 4A). Consistently, co-culture with mast cells significantly increased the percentage of S-phase BPH-1 cells, as showed in the flow cytometry results (Figure 4B). These finding suggested that infiltrating mast cells could promote BPH-1 cells proliferation.

### Mast cell-derived IL-6 plays a pivotal role in inducing BPH-1 cells proliferation

**Figure 5:**
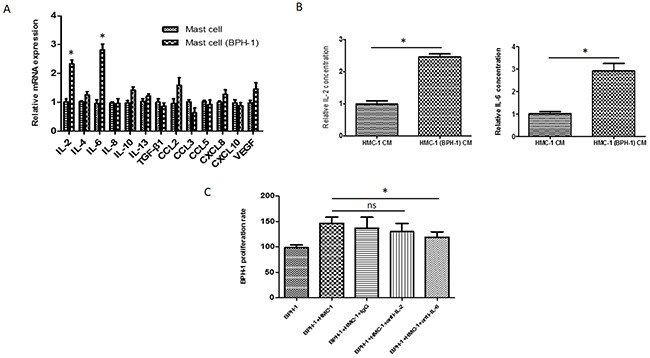
IL-6 as a mediator for mast cells induced BPH-1 proliferation **(A)** qRT-PCR analysis of cytokine/chemokine expression levels in mast cells 24 hours after culturing alone or co-culturing with BPH-1 cells. **(B)** ELISA analysis of IL-2 and IL-6 in the conditioned medium isolated from mast cells, or the co-culture of BPH-1cells and mast cells. **(C)** BPH-1 cells were co-cultured with mast cells in the presence of anti-IL-2/6 neutralizing antibody or IgG and assayed by MTT assay at 72 hours. *P < 0.05 by Student's t-test, ns: not significant.

Mast cell can release different mediators under certain circumstances to influence disease progression [23]. To find out which cytokine/chemokine plays a crucial role in mast cell-induced prostate epithelium cell proliferation, we adopted qRT-PCR to detect any potential factors that could be up-regulated in this co-culture system. We found that interleukin 2 (IL-2) and interleukin 6 (IL-6) mRNA were increased significantly in mast cells after co-culturing with BPH-1 cells, implying that the cross-talk of mast cells and BPH-1 cells may trigger the release of IL-2 and IL-6 (Figure 5A). We used enzyme linked immunosorbent assay (ELISA) to further verify the elevation of the IL-2 and IL-6 protein in the conditioned medium. As shown in Figure 5B, the concentration of IL-2 and IL-6 protein are much higher in the co-culture medium than in the control group.

To determine the role of IL-2 or IL-6 in the co-culture system, neutralizing antibody against IL-2 or IL-6 was added into the medium. The MTT assay unveiled that neutralizing against IL-6 (not IL-2) could partially reverse the effects of mast cell on BPH-1 cell proliferation (Figure 5C). These results from Figure 5A–5C, suggested that IL-6 is the key mediator in the process of mast cell-induced prostate epithelium cells proliferation.

### Mast cells enhanced BPH-1 proliferation via IL-6/STAT3/Cyclin D1 signaling

**Figure 6:**
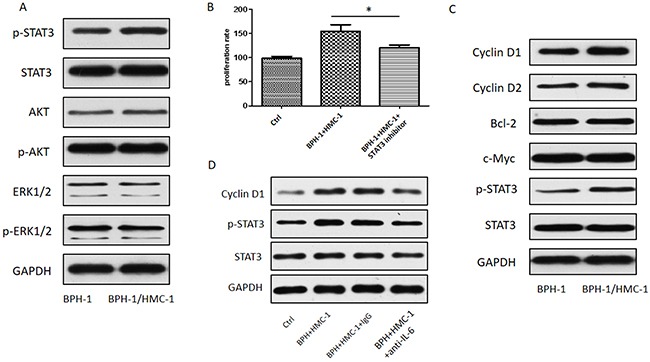
STAT3/Cyclin D1 signaling mediating mast cells induced BPH-1 proliferation **(A)** Western blot analysis of proliferation related protein kinases in BPH-1cells. **(B)** MTT assay for BPH-1 cells absence or presence of p-STAT3 inhibitor. Graphs shown are mean of three experiments. *P < 0.05 by Student's t-test. **(C)** Western blot analysis of STAT3 downstream genes related to proliferation in BPH-1 cells. **(D)** Western blot analysis of STAT3 and Cyclin D1 absence or presence of neutralizing antibody to IL-6.

To further dissect the mechanisms that mast cells promote BPH-1 cells proliferation, we detected the expression of JNK, ERK, and STAT3 by using Western blot. The results showed that mast cells could enhance p-STAT3 signaling in BPH-1 cells (Figure 6A). We next to examine the role of STAT3 signaling in mast cell-induced BPH-1 proliferation by co-culturing the BPH-1 cells with mast cells in the presence or absence of p-STAT3 inhibitor. As shown in Figure 6B, STAT3 inhibitor could significantly suppress the mast cell-induced BPH-1 proliferation. We further evaluated the downstream molecules of STAT3 pathway and found that Cyclin D1 was increased in BPH-1 cells after co-culture with mast cells (Figure 6C). To verify whether mast cell-derived IL-6 promotes BPH-1 cells proliferation through STAT3/Cyclin D1 signaling pathway, we then adopted an interruption approach to treat BPH-1 cells by using anti-IL-6 neutralizing antibody. The Western blot results indicated that blocking IL-6 partially reversed the STAT3/Cyclin D1 signaling (Figure 6D). Our findings suggest that mast cell derived IL-6 might promote BPH-1 cell proliferation through STAT3/Cyclin D1 pathway.

### Association between the infiltration of mast cells and clinical findings in BPH patients

We investigated the correlation between mast cell infiltrations into human BPH tissues and BPH patients′clinical findings (Table 1). We divided the BPH patients into two groups, with or without mast cells infiltrating, according to the IHC staining results. Of overall 111 BPH patients, mast cells were observed in 84 patients′ clinical specimens. Clinical data were collected to compare with each other between the two groups. The IPSS-Total was significantly higher in the mast cell infiltrating group compared to the group without mast cells infiltrating (20.1±8.66 VS 14.2±7.94; p=0.002). Similarly, IPSS-storage was associated with mast cell infiltration: patients with mast cell infiltrating presented higher IPSS-storage score.

**Table 1:** Patient characteristics and descriptive statistics

Variables	Infiltrated mast cells		P value
	With	Without	
Patients	84	27	
Age (y)	68.3±9.07	62.5±8.62	0.004
BMI(kg/m2)	25.3±5.46	23.8±3.62	0.185
IPSS-total	20.1±8.66	14.2±7.94	0.002
IPSS-voiding	9.44±4.31	9.58±4.72	0.886
IPSS-storage	10.2±4.29	5.52±3.07	< 0.001
QOL score	4.11±1.39	3.78±1.46	0.291
Total PSA (ng/ml)	6.13±3.72	4.95±2.19	0.122
TPV (ml)	64.9±26.1	52.3±19.4	0.023
TZI (%)	45.2±13.5	39.8±18.4	0.102
Qmax (ml/s)	8.04±5.47	7.66±3.05	0.732
Voided volume (ml)	213±149	238±143	0.446
PVR (ml)	20.4±7.95	19.2±9.67	0.519

BMI, body mass index; PSA, prostate specific antigen; IPSS, International Prostate Symptom Score; V, voiding subscore; S, storage subscore; QOL, quality of life; TPV, total prostate volume; TZI, transition zone index; Qmax, maximum flow rate; PVR, postvoid residual.

## DISCUSSION

BPH is a common urologic disorder among elderly males characterized by proliferation of the prostate epithelial and stromal cells. These pathologic changes result in enlarged prostate, bladder outlet obstruction, and lower urinary tract symptoms [24].

The etiology of BPH is still uncertain, but multiple factors may contribute to BPH progression. Chronic inflammation are known to play a crucial role in the pathogenesis of BPH [25]. The pathological analysis of BPH specimens showed increased immune cells infiltration and pro-inflammatory factors secretion occurred in the BPH tissues. Clinical evidences also supported the positive relationship between inflammation and BPH progression [26]. As multifunctional cells, mast cells can regulate inflammation through secreting different mediators. Mast cells have been observed in BPH specimen, implying mast cells are involved in BPH pathogenesis [11]. In this study, immunohistochemical results demonstrated that increased mast cells were present in the human BPH prostate tissues comparing to the normal prostate tissues. We investigated the interactions between mast cells and BPH-1 cells and found that BPH-1 cells could recruit mast cells and stimulate their activation. In turn, mast cells could promote BPH-1 cells proliferation through IL-6/STAT3/Cyclin D1 signaling pathway.

To study the interactions between mast cells and BPH-1 cells, we established an *in vitro* transwell co-culture system. We found the crosstalk between mast cells and BPH-1 cells could trigger the activation of mast cells and promote migration of mast cells. Considering that mast cells express several chemokine receptors, especially in inflammation, chemokines and chemokine receptors expressed in mast cells are likely to play a pivotal role in mast cell recruitment. Previous study reported that numerous mast cell-related chemoattractants like CCL5, CXCL12, tumor-derived peptides, transforming growth factor (TGF)-β isoforms, fibroblast growth factor (FGF), and platelet-derived growth factor could drive mast cells migration [27]. CXCL12, as one of the CXC chemokines, was previously shown to be involved in chronic inflammation, chemotaxis, and tumor development via its specific receptor CXCR4. Kryczek et al reported that tumor cells and stromal cells secreted CXCL12 were responsible for mast cells recruitment [28]. We herein adopted qRT-PCR to screen the expression of mast cell-related chemoattractants in BPH-1 cells. The cross-talk between mast cells and BPH-1 cells enhanced the release of CXCL12 from BPH-1 cells and increased the expression of receptor CXCR4 in mast cells. Importantly, blocking CXCL12 with its neutralizing antibody largely reversed BPH-1-induced mast cells migration. These findings suggested that CXCL12/CXCR4 axis may be the key factor that drive mast cell migrating to BPH prostate tissues.

In addition, while BPH-1 cells could trigger mast cell activation and cytokine release, recruited mast cells appears to promote BPH-1 cells proliferation. It has been reported that mast cells participate in a wide range of diverse biologic processes through secreting diverse mediators [23]. To dissect how mast cells enhance BPH-1 cells proliferation, we investigated a series of most reported cytokines or chemokines that are related to mast cell functions. The mRNA levels of IL-2 and IL-6 were up-regulated significantly in mast cells after co-culturing with BPH-1 cells. We further confirmed that the protein levels of IL-2 and IL-6 were increased in the co-culture medium using ELISA assay. However, it was IL-6, not IL-2, neutralizing antibody that could partially reverse mast cell-enhanced BPH-1 proliferation in the co-culture system. These findings implied that mast cells promoted BPH-1 proliferation mainly through secreting IL-6.

As a pro-inflammatory cytokine, IL-6 was identified to promote the development of BPH in previous study [29], which is consistent with our findings. To find out which pro-survival signaling pathway was responsible for IL-6 enhanced BPH-1 proliferation in our *in vitro* co-culture system, we applied Western blot assay to detect ERK, AKT, and STAT3 signals changing. The phosphorylated STAT3 increased significantly in BPH-1 cells after co-culturing with mast cells. STAT3, which is has been thought to be activated primarily by cytokines and growth factors, is an important transcription factor that regulates the expression of numerous genes, thereby contributes to various pathophysiological processes [30]. Therefore, we identified some common STAT3 downstream factors related to cell survive and proliferation, such as Cyclin D1, Cyclin D2, c-Myc, and BCL-2. In the cross-talk between mast cells and BPH-1cells, Cycllin D1 might play a key role in mediating STAT3 promoted BPH-1 proliferation.

BPH patients are faced with bothersome lower urinary tract symptoms (LUTS). The International Prostate Symptom Score (IPSS) is a widely used scale for detecting the severity of LUTS [31]. In this study, we found that mast cell infiltration in prostate tissues was positively associated with total IPSS and IPSS-S. These results further indicated that mast cells in the BPH tissues might play an important role in the BPH progression.

In summary, our study demonstrated that infiltrating mast cell could promote BPH epithelial cell proliferation through modulating IL-6/STAT3/Cyclin D1 signaling. Blocking mast cell migration or interrupting this newly identified signaling may help us choose better therapeutic strategies for BPH patients.

## MATERIALS AND METHODS

### Patients and clinical specimens

From 2014 July to 2016 October, BPH prostate specimens were collected from 111 patients who were diagnosed with BPH and received transurethral resection of prostate (TURP) in Xiangya Hospital, Central South University, Changsha, China. During the same period, we obtained normal prostate tissues from 16 patients with bladder cancer who received radical cystectomy. All these normal prostate specimens were examined by pathologists and turned out to be no hyperplasia evidence. Informed written consent was obtained from all patients. The current study was approved by the ethics committee at Xiangya Hospital of Central South University.

### Cell lines and cell culture

Human benign prostate epithelial cell line, BPH-1 cells were cultured in RPMI-1640 medium supplemented with 10% fetal bovine serum (FBS). HMC-1 cells were cultured in Iscove's modified Dulbecco's medium (IMDM) with 10% heat inactivated FBS. Cells were incubated in a humidified 5% CO2 environment at 37°C.

### Co-culture experiments and co-culture medium preparation

For the co-culture experiments, mast cells (1×10^5)^ were seeded in the upper 0.4 μm pore polycarbonate membrane inserts of six-well transwell plates containing BPH-1 cells (4×10^5^) in the lower chamber with RPMI-1640 medium containing 10% FBS for 48h. Then, cells were collected for further study; the co-cultured medium was collected and filtered with 0.22 μm filters (Millipore) for further experiments.

### Reagents and materials

GAPDH, p-STAT3 (Tyr 705), STAT3, JNK, p-JNK, ERK1/2, p-ERK1/2, Cyclin D1, Cyclin D2, Bcl-2, and c-Myc antibodies were purchased from Santa Cruz Biotechnology. The STAT3 inhibitor S3I-201 was purchased from Santa Cruz Biotechnology. Neutralizing antibodies to CXCL12, IL-2, and IL-6 were purchased from R&D Systems.

### Mast cells recruitment assay

The recruitment assay was performed in a 24-well transwell system with 8 μm pore polycarbonate membrane inserts (Corning). Approximately 1 × 10^5^ HMC-1 cells in 400 μl serum free medium were plated into the upper chambers. Conditioned media (CM) from BPH-1 cells or co-culture BPH-1/HMC-1 cells were added into the lower chambers of the transwells. After 6 hours, the HMC-1 cells migrated into the lower chamber medium were collected and the number of cells was counted with a hemocytometer (Bio-Rad TC20).

### Cell proliferation and cell cycle assays

Cell proliferation was measured using MTT assay. Approximately 5000 BPH-1 cells after co-culturing with HMC-1 cells or culturing alone were seeded into 24-well plates. After 72 h, 0.5 mg/ml of 3-(4,5-dimethylthiazol-2-yl)-2,5-diphenyltetrazolium bromide (MTT) (Sigma–Aldrich) was added to each well for 1 h and dissolved with DMSO. The absorbance at 570 nm was measured.

For cell cycle analysis, BPH-1 cells were co-cultured with HMC-1 cells or cultured alone for 48h, then collected, fixed with 70% ethanol at -20°C for 24 h, stained with 50 μg/ml propidium iodide and analyzed with a FACS Calibur.

### Enzyme-linked immunosorbent assay

The concentration of TNF-α, IL-2, and IL-6 in the conditioned medium was measured with ELISA kits for human TNF-α, IL-2, and IL-6 (DTA00C/D2050/D6050, R&D Systems) [32]. The assays were conducted following the manufacturers’ instructions.

### RNA extraction and quantitative real-time PCR analysis

Total RNAs were isolated from cells using Trizol reagent (Invitrogen, Grand Island, NY). Two μg of total RNA was applied for reverse transcription using Superscript III transcriptase (Invitrogen). Quantitative real-time PCR (qRT-PCR) was conducted using a Bio-Rad CFX96 system with SYBR green to measure the mRNA expression level of genes of interest. Experiments were repeated three times and GAPDH was used as internal control.

### Western blot analysis

Western blotting was performed as previously described [33]. Briefly, BPH-1 cells or mast cells were washed with PBS and then lysed in RIPA buffer. Quantified proteins were separated on 8-10% sodium dodecyl sulfate (SDS)-polyacrylamide gel electrophoresis (PAGE) and then transferred onto PVDF membranes. After blocking membranes using non-fat milk for 1 hour at room temperature, the membranes were incubated with specific primary antibodies (1:1000 dilution) overnight at 4°C. Tris-buffered saline plus 0.05% Tween-20 (TBS-T) was used to wash PVDF membranes. The blots were incubated with peroxidase-conjugated secondary antibodies (antirabbit or antimouse) for 1 hour and visualized using enhanced chemiluminescence system (Thermo Fisher Scientific).

### Immunohistochemistry

Human prostate tissues were fixed in 10% (vol/vol) formaldehyde in PBS and embedded in paraffin. The embedded tissues were then cut into 5-μm thick sections. The tissue sections were deparaffinized in xylene solution and rehydrated using gradient ethanol concentrations. Immunostaining was performed as described previously [33].

### Statistical analysis

All data/values were presented as mean ± SD from at least 3 independent experiments. Statistical analyses were performed with SPSS 17.0 (SPSS Inc., Chicago, IL). Differences in mean values between two groups were analyzed by two-tailed Student's t test. P values < 0.05 were considered statistically significant.
